# Community engagement for water reuse technologies for environmental health risk assessment and management

**DOI:** 10.2166/wrd.2026.112

**Published:** 2026-02-02

**Authors:** Amanda M. Wilson, Paula Kehoe, Kerry A. Hamilton, Paloma I. Beamer, Benjamin S. Wilfond, David B. Resnik

**Affiliations:** a Department of Community, Environment & Policy, Mel and Enid Zuckerman College of Public Health, University of Arizona, Tucson, AZ, USA; b San Francisco Public Utilities Commission, San Francisco, CA, USA; c Biodesign Center for Environmental Health Engineering, Arizona State University, Tempe, AZ 85281, USA; d School of Sustainable Engineering and the Built Environment, Arizona State University, Tempe, AZ 85281, USA; e Seattle Children’s Research Institute, Seattle, WA, USA; f University of Washington School of Medicine, Seattle, WA, USA; g National Institute of Environmental Health Sciences, National Institutes of Health, Research Triangle Park, NC, USA

**Keywords:** community engagement, ethics, implementation science

## Abstract

While there is an emerging consensus among scholars, academic researchers, and environmental advocates that community engagement is essential for effective environmental health risk assessment and management, robust engagement does not occur often enough. To help promote community engagement in environmental health risk assessment and management, we provide rationales and approaches. We illustrate our points with a case study concerning water reuse, as water recycling technologies expand across the United States and the world to address water scarcity and increased water demand, considering a community engagement continuum framework. Formal environmental risk assessment has always had a required component for public notification to seek comments, but it is arguably not meaningful community engagement, as it is often done after the risk assessment is completed (without input) and in response to contamination, without multi-directional community engagement. We show how effective multi-directional engagement of the community from the beginning of the risk assessment process can help to ensure that risk assessment and management are fair and effective. We also discuss some potential obstacles to effective community engagement concerning environmental health risk assessment and management and offer some recommendations for engineers, risk assessors, and policymakers.

## Graphical Abstract

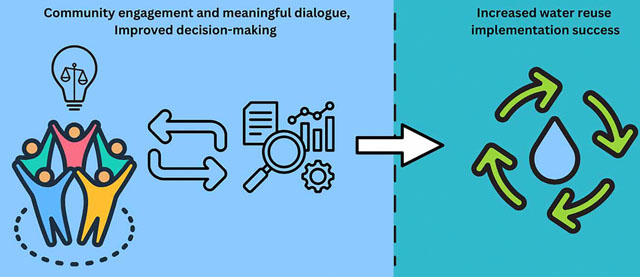

## INTRODUCTION

Societies face increasingly challenging environmental health issues that pose risk-risk tradeoffs (Frumkin 2016; Heida et al. 2022; Wilson et al. 2025a). That is, a technology or intervention that is implemented to reduce the risk of one outcome that may inadvertently increase the risk of another. One such example is water reuse, where water recycling technologies implemented to address increasingly severe threats of water scarcity (Fuller 2010; Gude 2017; Wang et al. 2024) may increase risks regarding chemical or microbial contaminants in public or even residential water supplies (Deshayes et al. 2015; Jahne et al. 2023). Water scarcity is a challenge worldwide, as new areas undergo desertification, with nearly 1 billion residing in drylands predicted to be impacted by water-related issues in the future (Shukla et al. 2019; Jain et al. 2024). It is inevitable that many communities will have to switch to water recycling or ‘reuse’ technologies, and the success of the implementation will depend on how well communities are engaged from the beginning.

‘Community’ can refer to specific groups of people within the general public or be referring to the general public as a whole, in some cases. In water reuse, this may mean those who are served by a water utility and will be directly impacted by new water reuse efforts at the utility level. However, it is helpful to think of community more broadly, as customers of utilities are not the only ones to be impacted by new water reuse endeavors. For example, regulatory entities, such as environmental departments of quality that permit implementation of direct potable reuse, may engage members of the public during the regulatory process in addition to engaging academic and research groups for input on technical aspects of rulemaking. Water utilities also play a key role in determining what that implementation will look like; whether rebate programs will be developed to encourage technology uptake; determination of the cost municipality-wide changes, like switches to direct potable reuse, etc. Research entities may also engage communities during research efforts, such as through surveys, interviews, and focus groups. This may be done independently or in partnership with regulatory entities, water utilities, or other organizations interested in evaluating water reuse implementation. Interaction across all of these partners, or ‘communities’, in planning and implementing water reuse leads to more successful implementation.

**Figure 1 |:**
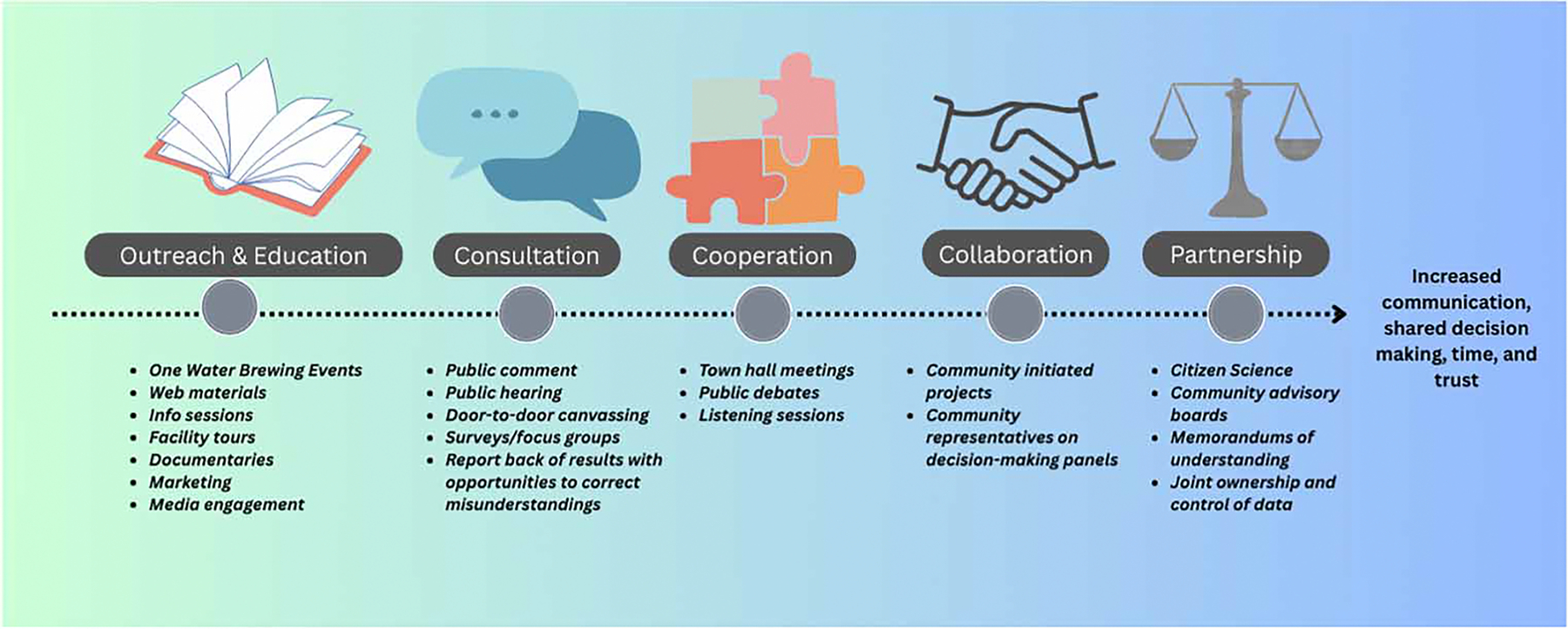
Examples of activities mapped to the community engagement continuum.

While these collaborative relationships could take many forms. Community engagement functions on a continuum, where efforts can increase according to the degree of substantive and meaningful communication, shared decision-making, time, and trust, as one advances on the continuum (Figure 1) (Key et al. 2019). Key categories of engagement activity, increasing along the spectrum, include (1) outreach and education, (2) consultation, (3) cooperation, (4) collaboration, and (5) partnership (Figure 1) (Key et al. 2019; Sanders Thompson et al. 2021). All aspects are important, where activities at the lower end of the continuum may include outreach, information sessions, facility tours, documentaries, and other media engagement. Activities at the higher end may include forming community advisory boards, developing memorandums of understanding with trusted community entities, conducting citizen science, or seeking community data sovereignty where communities own and control data originating from research on their community, specifically (Figure 1).

Across this continuum, community engagement for water reuse should ideally inform our understanding of how people value new water treatment technologies or interventions, especially when these are at the nexus of competing health outcomes. This may have implications for economic analyses regarding funds for technology interventions and the feasibility of achieving broad acceptance of water reuse technologies and the use of treated water. Community engagement is also needed in risk assessment and management to inform the risk scenarios that are explored and how human behavior parameters and assumptions are handled. For example, including knowledge from water utility customers about how people interact with new technologies or interventions reduces uncertainties in estimated risks. Human behavior data are vital for accurately modeling exposures and subsequent risks, which ultimately drive the needed performance levels of treatment technologies.

While community engagement with members of the public is important and is included in many environmental health efforts, the form in which engagement occurs is limited and may only be a recommendation as opposed to a required practice, depending upon the context. In the case of water reuse, there is guidance for how to engage communities focused on raising awareness, as opposed to creating avenues for the public to voice an opinion on the issues or make meaningful contributions to the development of the regulations. For example, the Water Research Foundation offers information on how to draft a message, developing informational material, building trust, and provides examples of engagement (e.g., round table discussions, facility tours, sponsoring local events) (The Water Research Foundation 2023). However, this information is only available for subscribers of the Water Research Foundation and focuses on increasing acceptability as opposed to involvement of communities in regulatory activity. Other organizations, such as Project Water Education Today (WET), offer guidance on engaging the public, but also not necessarily informing regulation development for water reuse.

Another form of commonly utilized community engagement is seeking public comment on regulations in development or holding public hearings. Public comment is also a required step in the Comprehensive Environmental Response, Compensation, and Liability Act (CERCLA) for risk assessment, where the public comment period follows the risk assessment drafting for a Superfund Site (Naval Facilities Engineering Systems Command – Engineering and Expeditionary Warfare Center no date). This is also true during Integrated Risk Information System (IRIS) assessments for individual chemicals regarding hazard characterization for environmental chemicals, where public comment on assessment drafts is elicited (U.S. Environmental Protection Agency no date). However, these are not always comprehensive and do not use modern methods. For example, it may be a newspaper ad or a post in the library as opposed to updated and likely more impactful approaches, such as emails and/or use of multi-media approaches including social media. These are often also only released in one language.

In the case of a water reuse example, the Arizona Department of Environmental Quality (ADEQ) opened public comment on direct potable reuse in Fall 2023 (Hahne 2023). Forms of engagement continued through Fall 2024, such as a virtual public hearing regarding a notice of proposed rulemaking, with plans to include this feedback in any rule revisions. This occurred before Notice of Final Rulemaking to the Governor’s Office (Arizona Department of Environmental Quality 2024). While these events are free and publicly available with the right technology access (i.e., internet access and a device with connectivity), the design of the hearing can limit the capacity of some of the public to engage, such as details on legal aspects of the new regulation and little information on how these new regulations are expected to impact water utility customers. Marketing approaches may also limit engagement, such as email alerts only to those who are signed up for listservs or postings on official websites that someone would have to seek out.

The aim of this perspective is not to criticize governmental or water utilities in their current practices. Rather, the aim is to explain the rationale and approach for effective community engagement, especially regarding customers of water utilities, to encourage its use by regulatory entities and water utilities. This will inform not only communication campaigns and efforts specifically intended to see public comment, but also will inform approaches for environmental health risk assessment and management. The concepts included in this piece are novel in the sense that commonly implemented community engagement strategies for advancing water reuse may not consistently engage the public or other parties in a meaningful way. We describe strategies meant to improve trust and successful outcomes when implementing new water reuse programs.

## THE EMERGENCE OF COMMUNITY ENGAGEMENT

For over 25 years, various scientists, engineers, ethicists, and governmental and non-governmental organizations have emphasized the importance of public and community engagement in the management of risks related to science, technology, industry, and the economy (Lavery et al. 2010; World Health Organization 2020; Resnik 2021). The National Academies of Sciences, Engineering, and Medicine (U.S.) (2016) defines engagement as ‘seeking and facilitating the sharing and exchange of knowledge, perspectives, and preferences between or among groups who often have differences in expertise, power, and values’ (National Academies of Sciences, Engineering, and Medicine (U.S.) 2016). Engagement, unlike education/outreach, goes in both directions, which means that all participants involved in the dialogue, including scientific experts and laypeople, can learn from each other. Engagement has been used as a tool for managing many different risks that affect communities and the public, such as risks related to genetically modified (GM) foods, waste disposal, urban development, clinical trials, and pollution (Dryzek et al. 2019; Resnik 2021). To be effective, engagement should be honest, transparent, respectful, inclusive, inquisitive, and deliberative. Engagement should occur early in deliberation, so that participants will have meaningful input into decisions or policies that affect them and will not perceive the outcome as a fait accompli (Lavery et al. 2010; Kolopack et al. 2015; Resnik 2018). While environmental risk assessment is often preemptive, it also has occurred in response to contamination resulting from decisions in which the public was not involved, such as the disposal of industrial waste in Love Canal from the 1920s to the early 1950s (Beck 1979). In the case of water reuse, there is still the opportunity to engage the public before the technology is broadly incorporated. Some useful strategies for engagement include town hall meetings, public debates, solicitation of public comments, focus groups, surveys, listening sessions at churches and civic organizations, and door-to-door canvassing of opinion (Kolopack et al. 2015; Committee on Genetically Engineered Crops: Past Experience and Future Prospects et al. 2016; Resnik 2018).

## RATIONALE FOR COMMUNITY ENGAGEMENT

There are three main reasons for engaging the community in risk management. The first is that community engagement promotes procedural justice concerning important decisions that affect members of the community (Resnik 2012, 2018; Neuhaus 2018). Procedural justice refers to the fairness of the deliberative process as opposed to fairness of the outcome of the decision (Rawls 2003). For example, one might say that a criminal trial was fair because it followed procedures for protecting the rights of the defendant even if one regards the outcome as unfair, e.g., that a person who was clearly guilty was exonerated. Because democracy involves the consent of the governed, it embodies procedural justice. Forms of democracy (i.e., direct democracy, representative democracy, and deliberative democracy) embody procedural justice (Gutmann 2004; Fishkin 2011). For example, if the government is deciding whether to locate a hazardous waste site in a particular area, principles of procedural fairness require that people who live in that area should have meaningful input into this decision (Resnik 2012).

The second reason for engaging the community is that this activity can improve the quality of risk management decision-making by yielding information that is useful for assessing risks, determining the acceptability of risks, and discussing the types of programs in place to manage that risk. In other words, community engagement can help to reduce the scientific and moral uncertainty regarding decision-making about risks. Risk management incorporates factual judgments concerning the probability that various hazards will cause harm and value judgments concerning the acceptability of risks in relation to potential benefits (Shrader-Frechette 1985, 1995). Because judgments about the acceptability of risks are based on moral, cultural, or social values, the community’s (or public’s) input is required. If scientific experts make decisions about the acceptability of risks without consulting the affected parties, such as communities or the public more generally, it is likely that their decisions will not reflect the interests, values, or opinions of those parties. To promote transparency and procedural fairness, it is important for scientific experts to explain to the public areas of agreement, disagreement, and uncertainty concerning the scientific evidence. For example, consulting with people who live near a potential site for a field trial of a water reuse project can yield information about the local ecology and human activities that could be useful in assessing the risks of the trial, as well as information about how and whether people affected by the trial think that the benefits are worth the risks. In discussing the trial with the community, scientific experts can convey what they know and do not know about the benefits and risks of the trial.

In addition to providing information concerning risk acceptability, there are other ways in which members of the public could engage in risk management efforts, such as voicing opinions about who is ultimately responsible for managing specific risks and what support may be needed for individuals to properly maintain those risks. For example, if purchasing a recirculating shower head, customers may have opinions about the type of information that should be disclosed by a landlord to a renter or from a seller to a buyer to inform renters or buyers about proper use of the showerhead in order to protect their health. Communication between potential customers and regulatory entities, utilities, and companies leading innovation in these areas is key to advancing the safe implementation of new water reuse technologies.

The third reason for engaging the community concerning risk management issues is to obtain support (or buy-in) for a technology, research program, public health intervention, or other activity that creates risks (Committee on Gene Drive Research in Non-Human Organisms: Recommendations for Responsible Conduct et al. 2016). The reason a specific technology or program is needed in the first place is sometimes due to cost or risk-related constraints. While fostering community/public support is not the primary reason for doing engagement activities, it is crucial for the implementation of risk management policies. If a community or public initially rejects a technology, research program, public health intervention, or other activity that is under consideration, then it may be very difficult to overcome this resistance, even if the activity offers the community/public important benefits. An instructive example of this problem is opposition to GM crops and foods in Europe. In the late 1990s, biotechnology companies began introducing GM crops in European countries without extensively consulting with the public about them. As a result, strong public opposition to these products emerged, and the European Union banned GM crops from 1998 to 2007 (Committee on Genetically Engineered Crops: Past Experience and Future Prospects et al. 2016; Resnik 2021).

## ILLUSTRATION: WATER REUSE CASE

While community engagement is important and applicable across many cases described above, we focus, herein, on water reuse due to (1) the far-reaching impacts of water scarcity and pressure to increase water reuse technology implementation; (2) the variability in voluntariness of these technologies and, resultingly, differences in perceived risks from the general public; and (3) variability in familiarity of these technologies and personal experiences in the general public, also driving variability in risk perception. Meaningful engagement can build both trust and reduce uncertainty in evaluating risks associated with water reuse technologies, making for smoother and safer implementation.

The far-reaching impacts of water scarcity across the globe have driven many communities to pivot to water reuse technologies at various scales, including direct potable reuse at municipalities (i.e., the treatment of wastewater to a high-quality standard making it safe for all uses, including drinking), down to recirculating showers or washing machines that result in water savings and operate at a household level. Due to varying levels of water reuse, the technologies and the risks they pose span a wide range of perceived voluntariness, which impacts how the risks are perceived (Slovic 2000). For example, if their water municipality uses direct potable reuse, customers do not have a choice in the water delivered to their home. If someone purchases a recirculating showerhead for their bathroom, this is a voluntary choice (but perhaps less so for the next owner). Voluntariness is important for informing risk perception, along with familiarity (Slovic 2000). If an individual receives direct potable reuse water at their home, they may not be knowledgeable about how the water was treated or where exactly it ‘came’ from before directly arriving at their tap. If an individual purchases a water reuse device for their home, it is possible they have done some research on the technology. Due to the breadth of water reuse applications, the risk perceptions can vary broadly across communities depending upon their familiarity with the technologies. Other factors shown to influence perceptions of drinking water, in general, include geographic region, household/neighborhood characteristics, household income, education level, sex, age, race and ethnicity, country of origin, sensory attributes, negative experiences with tap water, and availability of information about water (Colburn & Kavouras 2021). Risk perception complexities place a high importance on *successful* community engagement, where success is often equated to acceptance of water reuse and increased adoption of water reuse technologies.

### Engaging communities to increase trust

Risk assessments and management strategies often require judgments about exposure, about how much risk is acceptable, and what to do in the event that the anticipated risks are not acceptable. This requires a value judgment, specifically. If scientific experts make this judgment without including the right expert voices, potentially impacted industry leaders and trusted community leaders, it is possible that this could deteriorate trust between communities facing the risks and regulatory entities and water utilities. There may already be distrust due to a lack of knowledge about the overall system that leads to the water delivered to their residence. Communities may not know who is ultimately responsible for upholding the quality of water reuse treatment or how it is evaluated. In conversation with community members in Arizona about direct potable reuse, some commented on a general distrust of government and, therefore, a distrust in direct potable reuse water from a public utility, unless tested by an independent party (Wilson et al. 2025b). One participant commented on the fact that being asked what she thought about direct potable reuse was building trust (Wilson et al. 2025b):

‘And then like this also builds trust, like getting our opinion about using for public use. and like you know, like, I’m not a scientist. I’m not anybody special, but you’re still, like, want my opinion...’

Asking community members how they feel about water reuse implementation in a conversational, face-to-face format, as opposed to a seemingly random survey for a forum that is impersonal, overly technical, or containing legalese and technical jargon, may give rise to increased trust and cooperative communities that perceive water reuse implementation as a decision made *with* them as opposed to *for* them. We recommend forms of engagement that encourage dialogue, whether that is through interviews, focus groups, or public events, such as town hall meetings or listening sessions. These forms of engagement may be categorized as ‘cooperation’ or ‘consultation’ on the continuum, depending upon the level of interaction and dialogue. These forms of engagement are a step up from forms that may encourage less dialogue, such as web materials (outreach and education) or online marketing (Figure 1). Dialogue may also be more fruitful and impactful if guided by trusted community leaders. Getting input from community partners can leverage their trust for higher participation and bigger impact of community engagement events. Community partners in our water reuse context could just be public entities, such as libraries, or advocates who promote the health of their communities, such as *promotores* (Victory et al. 2022). These partners could also be organizations devoted to educating and advocating for sustainable water use, such as Project WET Foundation (Project WET: Water Education Today no date a, b), or a trade association, such as the Water Reuse Association (WaterReuse Association no date).

Conversations with community members can also inform their values around water scarcity: How important is it that we find a way to keep water affordable in a desert with an increasingly threatened water supply? What are the greatest considerations that impact how we weigh the risks posed by water reuse technologies vs. the risks posed by staying the course and running out of water? Where are the education gaps that, if addressed, would give people a more informed view on the topic?

### Engaging communities to reduce risk assessment uncertainty

One of the benefits of engaging communities in a meaningful way (i.e., in a way that encourages open and inclusive dialogue) is that the risk assessments used to inform regulations or guidance on treatment performance targets can better reflect real-world exposure scenarios. The general public is typically thought of as the primary community for engagement. For example, a risk assessment may estimate low ingestion volumes of water during bathing activities, from the perspective of a parent of small children who witnesses their child swallow mouthfuls of water nearly every bath time is considered. This may lead to a reconsideration of the scope of the risk assessment to address a worst-case scenario in which a child swallows mouthfuls of bath water on a nightly basis. Engaging with the public about scenarios of technology use broadens the potential list of exposure pathways to better understand variability in a case- or site-specific way.

Another community to engage to reduce uncertainty in how the technologies are mathematically modeled in the risk assessment may also include the companies making water reuse technologies for residential scales (i.e., individual home-owner/renter use). Knowing how the devices work has crucial implications for the accuracy of the risk assessment. For example, if a key assumption in the risk assessment is that water is stored between uses in recycling clothes washing machines, the estimated risk will be vastly different than if it is assumed that no water is stored, posing fewer opportunities for pathogen growth. Lastly, if it is learned that over half of a community would not be comfortable using direct potable water reuse for drinking but would happily use it for watering their lawn, edible garden, or for toilet flushing, perhaps a risk assessment includes weighing the risks posed by those activities as more important than drinking. It is clear from these examples that risk assessments require judgments (Shrader-Frechette 1995) about how conservative to be in protecting people from risks and what to do in the face of uncertainty about how the exposures occur. Behavioral information from those engaging in the risky activities in question increases the certainty in the risk assessments. Additionally, engagement within the risk assessment community is important to understand the biases held by risk assessors engaging in the risk assessments and to diversify experiences and knowledge to inform the risk assessment process.

## DISCUSSON

### Lessons for environmental health risk assessment and management

We have given examples of ways in which different forms of community engagement could be used across the community engagement continuum (Figure 1) to advance the implementation of water reuse technologies. Community engagement can and should come in a variety of modalities to garner broad reach across a community. To identify the most effective modalities, trusted community leaders should be engaged for insights into how communities receive information and are likely to engage in meaningful dialogue. Some unifying principles in how these efforts should be done include early, meaningful, and transparent involvement. The inclusion of early risk communication was recommended by the National Research Council, stating risk communication should be ‘early and sustained interchange’ (National Research Council 1990), consistent with the idea that more advanced efforts on the community engagement continuum increase in the level of meaningful involvement of community members in discussion and decision-making, with the highest form being shared power (Figure 1) (Key et al. 2019). DeShields & Pattanayek (2023) described the importance of representation of communities in decision-making processes who are impacted by current environmental health issues or who will face risks from new technology implementation (DeShields & Pattanayek 2023), such as that related to water reuse. Community engagement is integrally related to risk communication, risk assessment, and public health.

Effective risk communication includes addressing cultural factors, as this influences communication processes in general and can impact how health- or environmental-related messages are received (Lapinski & Oetzel 2021). Environmental communication, such as that related to water scarcity issues, can especially be challenging, due to the ‘interrelated nature of a globalized environmental reality’ (Takahashi et al. 2021). In guidance on cultural message tailoring related to interventions, Lapinksi & Oetzel (2021) reiterated the importance of community engagement as being on a ‘continuum with increasing levels of community involvement, decision-making, trust, and communication’. Effective risk and safety communication and community engagement are inextricably linked.

Some modern approaches to utility management include community involvement in the design, implementation, and evaluation of programs and policies. For example, the San Francisco Public Utilities Commission adopted a Community Benefits Policy that commits to these ideals (San Francisco Public Utilities Commission 2011). The policy includes language related to ‘culturally competent communication strategies to ensure that all stakeholders can participate in decisions and actions that may impact their communities’ (San Francisco Public Utilities Commission 2011). Guidance on how such approaches can be achieved will empower utilities to incorporate meaningful community engagement into ongoing and future efforts.

### Barriers to community engagement

While we illustrate the importance of community engagement for advancing water reuse and give some examples of what meaningful community engagement could look like, it does not come without its challenges. For example, a responsible party or individual is needed to organize, conduct, and coordinate the assessment with communication channels (e.g., media) for successful messaging about community engagement opportunities and outputs. These efforts require time, money, and expertise. It may also be counterproductive to successful implementation if the engagement is done in a way that triggers outrage or uncoordinated messaging from the media. In the Sandman model of community risk perception, outrage is more likely due to a variety of factors including the consequences are dreaded, the hazard is controlled by others, the exposure is unfair, and if the process is unresponsive (Chapman & Wutzke 1997). These may be pertinent to water reuse technologies at the utility scale due to lack of voluntariness and limited knowledge of the overall process in the case of a system failure. There also may be opposition from other parties with conflicting views, whether this be industry or political sources. Lastly, changes to community engagement may be slow due to general hesitancy to stray from what has always been done and regulations that are in place regarding specific types of engagement, such as public comment periods.

### Limitations and future scholarship

In this work, we have only explored water reuse generally. It is likely that community engagement will and should look different depending upon the type of water reuse application being addressed and in a community-specific context. More research is needed to inform best practices for engaging community members in meaningful dialogue about the implementation of water reuse technologies.

## CONCLUSIONS

Water reuse implementation is on the rise in the face of increasingly urgent water scarcity challenges around the world. As new technologies or systems are being introduced, the engagement of the public is paramount for success. In this opinion piece, we argue that there is room for advancing meaningful community engagement to support successful water reuse implementation. This primarily refers to the engagement of members of the general public, but can also include the adoption of a broader definition of ‘community’, where regulators, utilities, and the water reuse technology industry act as partners to collaborate on the safe and effective implementation of water reuse strategies. Luckily, there are other environmental technology examples throughout history that offer insights and provide lessons and perspectives on how to successfully gain the public’s trust and support. Regardless of the environmental context, community engagement occurs on a continuum, and we argue for more meaningful engagement (i.e., opportunity for dialogue and partnership) from the beginning.

## Data Availability

All relevant data are included in the paper or its Supplementary Information.

## HIGHLIGHTS

- More guidance is needed for utilities on how to meaningfully engage communities.
- We use a case study to demonstrate meaningful community engagement strategies.
- Engaging communities improves risk assessment and decision-making.
- Engagement should be early and meaningful, encouraging dialogue.
